# Patient perspectives on their outcomes from strabismus surgery undertaken for psychosocial reasons

**DOI:** 10.1038/s41433-024-03189-9

**Published:** 2024-06-21

**Authors:** Gemma E. Arblaster, Helen Davis, David Buckley, Sarah Barnes

**Affiliations:** 1 School of Allied Health Professions, Nursing and Midwifery, University of Sheffield, Sheffield, UK; 2 Orthoptic Department, Ophthalmology, Sheffield Teaching Hospitals NHS Foundation Trust, Sheffield, UK; 3 School of Medicine and Population Health, University of Sheffield, Sheffield, UK

**Keywords:** Outcomes research, Health care

## Abstract

**Background:**

Strabismus surgery undertaken for psychosocial reasons aims to align the eyes in a straighter position, reduce the psychosocial symptoms experienced and improve health related quality of life (HRQoL). Greater evidence of the postoperative outcomes in adults undergoing strabismus surgery for psychosocial reasons is required to inform funding and commissioning decisions about strabismus surgery.

**Methods:**

Semi-structured interviews were conducted with adults who had previously undergone strabismus surgery for psychosocial reasons to explore their perceptions of their postoperative outcomes. Maximum variation sampling was used to recruit males and females, younger and older participants. Interviews were transcribed and analysed using thematic analysis following the principles of grounded theory.

**Results:**

Thirteen adults were recruited and interviewed, mean 12.2 months postoperatively (range 4.5–20 months). Participants reported a range of improvements in vision, task performance, physical symptoms and confidence and emotions. Some worsening of physical symptoms was reported.

**Conclusion:**

Despite undergoing strabismus surgery for psychosocial reasons, a range of improvements in vision, task performance and physical symptoms were reported by adult patients postoperatively, in addition to the expected improvements in confidence and emotions.

## Introduction

Strabismus surgery may be undertaken to align the eyes in a straighter position and improve psychosocial symptoms, in the absence of measurable binocular single vision (BSV) or visual symptoms, such as diplopia and/or confusion [1]. Despite strabismus surgery for psychosocial reasons being considered as highly beneficial for patients [2], improving quality of life and being highly cost effective [2, 3], some areas of England have withdrawn national health service funding for this type of surgery. There was concern that not enough patient benefit was proven in those without expected functional visual gains from surgery [4]. Psychosocial symptoms in strabismus include being more likely to suffer with anxiety and clinical depression [5], psychological problems [6], receiving ridicule [6], low self-esteem and self-confidence [7–9], less likely to gain employment [10], avoiding social situations or activities [8–11], difficulty making eye contact [8, 9, 12], and problems with interpersonal relationships [6, 8]. Patients with strabismus report poor visual function and health related quality of life (HRQoL), those without diplopia report greater psychosocial concerns [13].

Strabismus surgery can significantly improve psychosocial symptoms [1, 7–9, 12, 14–16] and other aspects of patients’ lives [17, 18]. Whilst all patients with strabismus can gain improvements in measures of anxiety, depression, HRQoL, social avoidance, daily functioning and psychological adjustment after strabismus surgery, it is those with psychosocial symptoms that gain most improvement [13, 14, 18, 19]. To help inform clinical and commissioning decisions [4], greater evidence of postoperative outcomes in patients with strabismus and psychosocial symptoms is required. This qualitative study aimed to explore the patients’ perspectives of outcomes from strabismus surgery undertaken for psychosocial reasons.

## Materials and methods

Following ethical and local approvals (REC and HRA 17/NW/0561) participants were recruited from Sheffield Teaching Hospitals NHS Foundation Trust to participate in semi-structured interviews. All were adults who had previously undergone strabismus surgery for psychosocial reasons, able to give informed consent and complete an interview in English. The study aimed for a diverse maximum variation sample of participants across age (younger 18–35 years old/older 36 years and above) and gender (male/female). Prior to the interview all participants were given a participant information sheet, had the study explained to them, an opportunity to ask questions and a choice of interview date and location. Individual written informed consent was taken before their interview.

During the interviews participants were invited to speak about their experiences and perceived outcomes from strabismus surgery. Open questions were asked about positive and negative changes, or no change postoperatively. Questions included vision, task performance, activities and daily life. Broader participant descriptions of experiences were not discouraged and participants were encouraged to expand on answers given and give examples. Participants were invited to add information at the end of the interview.

All interviews were conducted by GA and audio recorded. Field notes were made during and after interviews. All interviews were transcribed verbatim (Express Scribe Transcription Software, version 6.10 ©NCH Software) as soon as possible after data collection. Interviews were listened to multiple times to ensure transcription was accurate and to enable familiarisation with the data. Transcripts were anonymised and stored in NVivo (QSR NVivo 10). Qualitative findings were analysed using thematic analysis following the principles of grounded theory [20]. A coding frame was developed independently (GA and SB) and a final coding frame agreed following discussion. The final coding frame was applied to all transcripts. The codes were used to organise and display the information in NVivo. Constant comparison was used to ensure there was simultaneous data collection and analysis, looking for emerging themes. The ‘one sheet of paper’ technique was used to look at higher level themes that emerged from the data, and to help search for connections and patterns within and across codes and themes. Data collection and analysis proceeded to achieve data saturation, when no new codes or themes emerged from the data.

## Results

Thirteen participants were interviewed, younger females (*n* = 4), older females (*n* = 3), younger males (*n* = 2) and older males (*n* = 4) (Table 1). All had undergone strabismus surgery for psychosocial reasons and were mean 12.2 months postoperatively (range 4.5–20 months). All had no diplopia and no measurable BSV, pre or postoperatively. All participants demonstrated suppression on sensory fusion testing and had at least perception of light vision in their poorest seeing eye.

**Table 1:** The clinical characteristics of the participants (*n* = 13).

Participant	Age gender	Postoperative diagnosis	Postoperative time (months)
1	18–35 F	Residual HT & XT	11
2	18–35 F	Residual ET (Nr) & consecutive XT (Dist)	20
3	36+ F	Residual ET & HoT with accommodative element	17
4	18–35 F	Residual ET with accommodative element	15
5	36+ F	Residual ET & DVD	14
6	36+ F	Residual ET & HoT & pseudoptosis	15
7	36+ M	Residual ET & HoT	9
8	36+ M	Residual XT	10
9	36+ M	Orthotropia	17
10	18–35 M	Residual consecutive XT & HoT	9
11	18–35 F	Residual ET	4.5
12	18–35 M	Consecutive ET	5
13	36+ M	Consecutive ET	12

*F* female, *M* male, *ET* esotropia, *XT* exotropia, *HT* hypertropia, *HoT* hypotropia, *DVD* dissociated vertical deviation.

Four main themes emerged from the analysis of the interviews: vision, task performance, physical symptoms and confidence.

### Vision

Nine participants described improvements in their vision postoperatively (Table 2). With both eyes open focussing was reported as better, allowing improved and easier performance of tasks under natural viewing conditions. Vision was described as sharper and clearer, leading to being able to notice more of their surroundings. Improved vision was reported to be beneficial when using screens, performing near activities such as artwork or cooking, driving, walking around, reading signposts and during social activities. Improved vision was reported as less blurred, more natural, easier to see, more central, straighter and more comfortable. Improved vision enabled greater levels of concentration, longer concentration and being able to perform better at work. Participants reported feeling able to tell that the strabismic eye was in the right place and being able to make more accurate judgements about the position and alignment of objects. Participants described feeling less confused, less distracted by strabismus, or less like their strabismus was getting in the way. Having less confusion was perceived to be due to looking through both eyes postoperatively. Four participants reported improved vision specifically in busy environments.

**Table 2:** Quotes that illustrate the theme vision.

Vision	
Clearer vision	*“I can definitely focus more now that I’ve had my squint surgery, on what I’m doing at my desk, on my computer” Participant 004, Female, 18–35 years*. *“everything since the operation I’ve been absolutely pleased with, yeah everything, it’s just made it a lot better… you’ve got a lot more, somehow, clearer view, everything’s straighter” Participant 007, Male, 36+ years*. *“yeah, 100% yeah, it just feels different, it just feels better, yeah, not as confused” Participant 009, Male, 36+ years*.
Peripheral vision	*“it’s like being more aware of your surroundings… it helps you feel a bit more safe… I can see more of my surroundings, so you can be a bit more alert… just a bit better and a bit more safe” Participant 002, Female, 18–35 years*.
Using the strabismic eye	*“I was trying to use my right eye a lot harder than my left eye, but now I can use them both together, so it’s a lot easier to focus on what I’m doing and work, my emails that I’m reading, so that’s good” Participant 004, Female, 18–35 years*. *“I don’t use both my eyes together… you do feel like… it’s easier to use it a bit more now…. I would say more to the side, I mean it is definitely a lazy eye, it’s still definitely a lazy eye, it doesn’t like being used very often, so I use my other one, but… when I do use it… it is alright, it’s a lot better” Participant 006, Female, 36+ years*. *“I don’t think I’ve done it (closing one eye) as much as I used to, I still do it now and then, if I’m getting a headache” Participant 012, Male, 18–35 years*.
Descriptions of unchanged vision	*“there’s no change in my vision, but it’s helped me to see a bit better, because it’s not going out and… like confusing, making it a bit blurry… it’s made that better” Participant 001, Female, 18–35 years*.
Adaptation postoperatively	*“I did have some headaches but… I suppose that’s to be expected, but that was also my brain getting used to looking through that eye” Participant 011, Female, 18–35 years*.

Five participants reported improved eye movements, including being able to look further into different positions of gaze, more comfortable eye movements and the eyes moving together more. An increased field of peripheral vision was described by seven participants. This was perceived as having better, clearer or more vision. The impact of a greater field of peripheral vision was reported as being able to see trip hazards or people at the side, being more aware of personal surroundings and feeling safer. Requiring less head movement to see to the side was reported by four participants and was described as a direct consequence of having greater peripheral vision postoperatively. One participant described losing panoramic vision following surgery for exotropia made his vision more normal.

Despite having no measurable BSV postoperatively, two participants reported using their eyes together, leading to improved focussing. Four participants reported they were using their strabismic eye more postoperatively, including taking up fixation with the strabismic eye quicker, having greater peripheral vision and needing less head movement to see to the side. This was perceived as happening automatically, leading to improved vision, but not binocular vision. Three participants described better vision in their strabismic eye postoperatively. Others reported gaining control over their strabismus and less strabismic eye closure, particularly during periods of concentration.

One participant described visual aspects that were better (blur) and worse (vision). Three participants reported no visual change postoperatively, however two gave examples of improved vision during their interviews. Changes in vision may have been subtle, of little benefit, or may represent variation in interpretation of ‘vision’. Participants reported varying periods of adaptation postoperatively. Some were aware of immediate visual improvements, yet others described headaches or bumping into things during their early postoperative period.

### Task performance

Improved task performance was reported by seven participants postoperatively (Table 3). Driving was the most commonly reported improved task. Seven (out of eleven) drivers reported driving was improved or easier. Other reported changes related to driving, including greater confidence when driving and vision being improved, clearer, more in focus, using the strabismic eye more, having greater peripheral vision and needing to use less head movements to look to the side.

**Table 3:** Quotes that illustrate the themes task performance and physical symptoms.

Task performance	
Driving	“*mainly when you’re driving and things like that, it’s better when you’re looking round and things… you do realise the difference… when you’re driving and… looking round corners or looking to the left and using that eye more, that’s where you sort of notice it a bit better” Participant 006, Female, 36+ years*
Work	*“I was always making mistakes… but now, I don’t, it’s very rare I have any mistakes at all…. (in my job) you have to work fast” Participant 010, Male, 18–35 years*.
Near tasks	*“now my judgement is a lot better…. if I was trying to thread a needle… I would miss it totally, but now I’m looking through my bad eye I can, I’m getting the judgement right on the hole and getting it through, and not have to like use just one eye to do it, I’m using both” Participant 011, Female, 18–35 years*.
Balance	*“I used to suffer with balance loss, because both my eyes… didn’t focus together, I was a bit like falling over or a bit clumsy, but now they work together I don’t think… it’s that bad, because my eyes are focussing together on something” Participant 004, Female, 18–35 years*.

Physical symptoms	
Headache	*“it’s a lot better, no more headaches, no more squinting, because of my eye… I was holding my eye to try and get the pain away… now there’s no more pain any more or anything” Participant 011, Female, 18–35 years*. *“the headaches have got worse though, so that’s a negative… the headaches are definitely worse, but I don’t know why that is” Participant 012, Male, 18–35 years*
Pulling sensation	*“if I read for quite a while, you could feel it pulling… you could feel something was dragging it to the side, that’s what it felt like…. it still pulls a little bit, but nowhere near as bad as it did, and I can read a bit longer now” Participant 009, Male, 36+ years*

Postoperatively the ability to work and perform specific tasks, such as operating tills or machines, at work was described as improved. Improved work ability included making fewer mistakes, being better at their job, working for longer, being able to work harder, needing to take less rest breaks and having improved concentration at work. Improved work ability was associated with having improved vision, straighter eye alignment, the strabismic eye drifting less, greater confidence in work abilities and greater confidence interacting with people at work. Improved performance at work was also associated with postoperative improvements in using computers and screen-based devices. Participants reported being able to work for longer on the computer, doing more and harder work, improved focussing on computer-based tasks, and greater visual comfort when looking at screens.

Near tasks and practical daily activities were reported as improved postoperatively. Participants reported improved reading and tasks such as putting keys into door locks, wiring in dim lighting, home DIY, cooking, drawing and carpentry. Near task improvements were reported as performing tasks for longer, more accurately, with fewer breaks, less eye strain and less frustration. Improved vision was associated with improved near task performance, including improved focussing, using both eyes, having improved judgement of position and needing to close one eye less. Despite having no measurable BSV, improvements were reported in tasks involving judgments of depth and the position of objects in space, such as navigating steps or obstacles, picking up objects, threading and three-dimensional computer games.

Improved balance and less dizziness, clumsiness and bumping into things were reported postoperatively. Improved balance was associated with improved focussing with the strabismic eye and feeling like the eyes were working together. Less dizziness was associated with the strabismic eye drifting less.

### Physical symptoms

After postoperative healing, participants described mostly improved physical symptoms, which they attributed to straighter eye position and the eye no longer turning, feeling tight or pulling (Table 3). Seven participants described completely resolved or improved eye pain, strain, discomfort, tiredness and epiphora postoperatively. Postoperatively headaches were reported as resolved or improved by six participants and worsened in one participant. One participant described worsened eye sensitivity and epiphora postoperatively. Having improved or resolved physical symptoms was associated with improved vision, closing the strabismic eye less, the eyes feeling more relaxed, improved concentration and being able to read for longer.

Participants reported improved physical symptoms during specific activities such as work, driving, near tasks and reading, or when tired or unwell. Participants associated improved physical symptoms with improved task and work performance, such as being able to read or work for longer, needing to take fewer rest breaks and no longer needing time off work (sick leave).

### Confidence and emotions

Improved eye alignment postoperatively was described as making a big impact on the lives of all the participants, leading to them feeling better and no longer worrying about their eyes and eye position (Table 4). Twelve participants described feeling happier, better in themselves, more confident and less stressed, anxious or worried postoperatively.

**Table 4:** Quotes to illustrate the theme confidence and emotions.

Confidence and emotions	
Self-confidence	*“it’s miles better, basically everything about it has just improved my life, I feel a lot more comfortable about things, more relaxed, rather than really tense all the time” Participant 009, Male, 36+ years*.
Confidence communicating with others	*“I found it a lot easier to talk to people in person and communicate with people” Participant 002, Female, 18–35 years*. *“I’m more willing to go for an interview, whereas before… I’d stay in the same job… because I’m getting older I want a better career and that’s holding me back (referring to strabismus), because I just knew… if they’d see my eye I’m thinking they’re not going to want to employ somebody like that… so, (postoperatively) I’ve recently applied for uni” Participant 011, Female, 18–35 years*.
Confidence in vision	*“I wouldn’t have applied for that kind of job, anything to do with driving, but now I would, I’m confident to think that my eyes are good enough to do that kind of thing” Participant 008, Male, 36+ years*.

Improved interactions and communication with people was reported by all participants. Examples included being able to go out in public, look at people, make eye contact and have face-to-face communication. Socially participants reported feeling less embarrassed, more relaxed and confident when meeting new people, socialising more, fitting in and no longer avoiding social situations or communication with people. Additionally, in a work environment participants reported being given more face-to-face opportunities and being better at their job, as they were perceived to be friendlier, less rude or more communicative.

Participants described no longer receiving comments about their strabismus or being treated differently. The ability to look at another person and communicate with them were described as important factors in postoperatively having confidence to put themselves forward for opportunities such as applying for a new job, going for a job interview, applying for university and starting a new career. Increased confidence in vision and performing tasks such as driving and work abilities were also reported. Having greater confidence in their abilities, eyes and vision were significant factors in feeling able to drive, work more and try new activities.

## Discussion

Improvements in vision, task performance, physical symptoms and confidence were reported following strabismus surgery undertaken for psychosocial reasons in adults. Worsening of physical symptoms (headache, eye sensitivity and epiphora) postoperatively were much less common than participants reporting positive outcomes after surgery.

### Vision

Improved VFQ-25 scores [14] and improved binocular summation [21] have been reported postoperatively, with greater improvements in binocular summation reported in later onset and larger strabismus, [22] and better postoperative alignment [21]. Improved binocular summation has been described as a functional improvement in vision [21] and it may explain the improved vision and reduced need for monocular eye closure [22] reported in this study.

Despite suppression on clinical testing, some participants described looking through both eyes at the same time or feeling that they were using their eyes together as a pair. This may be due to an expanded field of vision with both eyes open [23] or suppression of a smaller area with a smaller deviation [24]. It may also be due to a change in the contribution of the apparently suppressed eye which has been demonstrated during visual field sensitivity [25], saccade [26], mobility [27], prehension [28] and motor skills [29] tasks.

Descriptions of improved peripheral vision, being able to see more and use less head movements to see may relate to an expanded visual field postoperatively [23]. Improved eye movements, being able to look further into different positions of gaze, more comfortable eye movements and the eyes moving together more may be related to improved ocular alignment or improved physical symptoms. In children (with and without BSV) Bucci et al. [30] measured reduced saccade disconjugacy postoperatively, but no significant change in saccade accuracy, mean peak velocity or post saccadic drift. This improvement was described as improved binocularity, however it may have been a consequence of recording eye movements (saccades) in eyes that were aligned and no longer had large angle strabismus.

### Task performance

Improvements in tasks and daily activities following strabismus surgery for psychosocial reasons have been reported previously using questionnaires [7, 15, 17]. Whilst details of these tasks or activities have not previously been explored, absent stereopsis has been associated with poorer prehension [31], and poorer and absent BSV associated with worse task performance [29, 32]. In this study, driving was reported as improved and was associated with improved vision. Driving with normal BSV has been compared to esotropia with suppression. Stereopsis led to improved slalom driving at intermediate distances, however participants with suppression were able to estimate the relative position of two cars better than those with BSV [33].

### Physical symptoms

Physical symptoms [34], physical discomfort and eye fatigue [11] due to strabismus, have been reported less commonly than psychosocial symptoms. In this study most participants described improved physical symptoms postoperatively, which was similar to improved eye strain reported by adults without diplopia [35].

### Confidence and emotions

All participants reported postoperative improved eye alignment made a large and significant impact on their lives, which is similar to reports of improved psychosocial factors, QoL and HRQoL postoperatively [1, 7–9, 12–14, 18, 19], even when BSV was not achieved [16]. Postoperatively participants reported feeling better at their job and having more or better work opportunities, similar to the findings of Ghiasi et al. [9]. Improved confidence postoperatively also led to participants putting themselves forward for education, work and career opportunities, which is similar to ‘changing plans for the future’ postoperatively [8, 12]. In this study being able to look at and communicate with people, and no longer receiving negative comments, attitudes and behaviours from others were important factors in gaining improved self confidence postoperatively. In addition, improved confidence in vision and abilities were associated with feeling able to work more, perform tasks, drive and try new activities, which is similar to the outcome ‘trying new or previously avoided activities’ highlighted by others [7–9, 12].

It is acknowledged that this study has limitations. A small number of participants were recruited (*n* = 13) and it is possible that more participants may have enhanced the data, however, it was felt that data saturation had been reached. Purposive sampling ensured a range of participants were recruited and semi-structured interviews allowed detailed and rich information to be gathered. A potential limitation was interviews were postoperative only, relying on recall, but the postoperative time point was variable. A narrower range of postoperative interview time would have been more standardised. During the interviews participants were invited to talk about a range of different topics and had the opportunity to add information and include different experiences. Open, ‘non-leading’, standardised questions were asked to avoid introducing bias and demand characteristics. However, it is acknowledged that implicit bias can occur when interviewing. An independent interviewer may have encouraged more varied responses.

## Conclusion

Adults who have undergone strabismus surgery for psychosocial reasons can report a number of improvements in their vision, task performance and physical symptoms, in addition to the expected improvements in confidence and emotions. Participants reporting worsening symptoms or negative outcomes from surgery were much less common than positive outcomes from strabismus surgery.

## Summary

### What was known before

- In the absence of binocular single vision and visual symptoms strabismus surgery may be undertaken for psychosocial reasons.
- Strabismus surgery for psychosocial reasons aims to align the eyes and reduce psychosocial symptoms caused by having strabismus.

### What this study adds

- Adults report greater postoperative improvements from strabismus surgery undertaken for psychosocial reasons than are measured clinically.
- Adults can report improved vision, task performance, physical symptoms and confidence postoperatively.

## Data Availability

The anonymised data analysed during the current study are available from the corresponding author on reasonable request.
